# Guiding principles for health equity in oncology: insights from patient organizations from the Middle East and Africa

**DOI:** 10.3389/frhs.2025.1560116

**Published:** 2025-10-17

**Authors:** Sawsan Al-Madhi, Lauren Pretorius, Emad Shash, Belma Kurdoglu, Ahmad Rabea, Atlal Abusanad, Hani Nassar, Hamida Kettab, Benda Kithaka, Khaled Abdel Aziz

**Affiliations:** ^1^ AlignnEficient Health Consultancies, Dubai, United Arab Emirates; ^2^ Campaigning for Cancer, Johannesburg, South Africa; ^3^ Medical Oncology Department, Shefaa Al Orman Comprehensive Cancer Center, Luxor, Egypt; ^4^ Medical Oncology Department, National Cancer Institute, Cairo University, Cairo, Egypt; ^5^ Cancer Survivors Association, İstanbul, Türkiye; ^6^ Baheya Cancer Center, Cairo, Egypt; ^7^ National Cancer Institute, Cairo University, Cairo, Egypt; ^8^ Najia Community for Survivors and Patients with Breast Cancer, Jeddah, Saudi Arabia; ^9^ Barbara Nassar, Beirut, Lebanon; ^10^ El Amel Association, Médéa, Algeria; ^11^ Kilele Health Association, Nairobi, Kenya; ^12^ Mersal Cancer Center, Cairo, Egypt

**Keywords:** health equity, patient-centered care, patient advocacy groups, health disparities, Middle East and Africa (MEA)

## Abstract

This paper explores the role of patient advocacy in fostering patient-centered care (PCC), empowerment, and equitable access to healthcare in the Middle East and Africa (MEA) region. Despite the vital contributions of patient advisory groups (PAGs) advocating for patients’ rights and helping informed decision-making, implementation of PCC encounters challenges such as cultural variances, language barriers, and general lack of understanding of concepts. A consensus-based Health Equity Steering Committee for the MEA PAG convened a virtual meeting, to identify current practices and gaps in patient-centric approaches in oncology. This discussion highlighted the critical role of collaborations across various sectors to enhance knowledge sharing, access to treatment advancements, and manage societal stigma. A significant gap was observed in data handling by patient organizations, underscoring the need to strengthen data analytics capabilities to improve access programs. Recommendations focused on strengthening collaborations, enhancing data analytics, and integrating patient perspectives into healthcare planning.

## 1 Introduction

The World Health Organization (WHO) defines equity as the absence of unjust, preventable disparities among different societal groups based on social, economic, demographic, or other forms of inequality, including sex, gender, ethnicity, disability, or sexual orientation (1). Health equity approaches in cancer care are necessary to ensures that every individual, regardless of socioeconomic status, geography, or other factors, has equal access to early detection, effective treatment, and supportive services, thereby minimizing disparities in outcomes and maximizing the potential for successful management (2).

### 1.1 Overview of cancer in MEA

Cancer has become a major health issue in the Middle East and Africa (MEA) region, with 9 countries, including Algeria, Egypt, and Saudi Arabia, seeing its rise from the third to the second leading cause of death between 2000 and 2019, accounting for 10%–13% of deaths (3). The incidence of new cancer cases per 100,000 individuals surged significantly, ranging from 10% to 100% with a projected increase until 2040, ranging from 27% in Egypt to 208% in the United Arab Emirates (3). In the MEA region, significant barriers impact cancer care, including cultural, educational, and socioeconomic factors, as well as health service capacity. A study of Ghana's health system reveals that specialized cancer care is concentrated in urban areas, leaving rural regions with insufficient training and specialists, leading to delays in diagnosis and treatment. Additionally, cultural factors such as distrust in formal healthcare and a preference for traditional medicine contribute to delays in seeking conventional cancer treatments (4). In Jordan which has one of the most advanced health care systems in the MEA region, socioeconomic disparities have resulted in over a quarter of the population lacking health insurance, affecting access to cancer care (5). This issue is particularly severe for Syrian refugees, who often present with advanced-stage cancer due to delayed diagnosis. Meanwhile, patients in Kenya and other low- and middle-income countries (LMICs) frequently face advanced-stage cancer diagnoses due to factors such as high costs and inadequate knowledge among healthcare workers about early cancer signs (6). Overall, disparities in cancer care in the MEA region highlight a critical need for systemic changes to address these inequities and ensure equitable access to cancer treatment and prevention.

A patient-centric approach significantly enhances health equity by addressing care disparities and fostering patient empowerment. It focuses on prioritizing patient needs, preferences, and experiences throughout the healthcare continuum (7). It also promotes collaboration among healthcare providers, researchers, policymakers, and patients, ensuring that healthcare interventions are aligned with patient needs (8). Engaging with patients not only drives innovation but also ensures that their perspectives are integrated into the drug development process from the beginning (7, 9).

In contemporary, equity-oriented health systems, patient advocacy embodies core principles of patient-centred care (PCC), including respect for patient needs, shared decision-making, and access to timely, appropriate services. PCC operates on approach of health equity by systematically reducing barriers to care and ensuring that services are designed around patient preferences and lived experience. Both through individual efforts and collective initiatives, patient advisory groups (PAGs) are crucial in enhancing patient representation, advocating for patients' rights, and promoting informed decision-making processes within healthcare systems (10). However, the implementation of PCC in the MEA region faces challenges such as cultural variances, language literacy barriers, healthcare provider behaviors, and a general lack of understanding about the concept of PCC (11). Many Middle East and North Africa (MENA) countries have pledged to pursue universal health coverage (UHC) with an aim to provide access to quality essential health services without risk of financial hardship to everyone. In December 2020, the UK Academy of Medical Sciences and Egypt's Academy of Scientific Research and Technology convened a virtual, multi-stakeholder meeting to review UHC advances across the region and to define how research could accelerate further progress. Precedent from such meetings suggest that structured, multi-stakeholder deliberations can help identify priority gaps and actionable agenda (12).

A consensus-based advisory board committee, comprising members from PAGs across the MEA region, convened to identify the current practices and gaps pertaining to health equity within patient-centric approaches for the MEA region in the oncology space. The primary objective was to discern the challenges faced by PAGs in the region concerning healthcare access, with a specific emphasis on elucidating the barriers obstructing equitable healthcare delivery and hindering the adoption of patient-centric practices. This paper presents a consensus-based discussion on health equity in oncology among PAGs from the MEA. It identifies current practices and gaps within patient-centric approaches across the region and summarizes the key issues encountered by PAGs. Additionally, it aims to provide actionable recommendations and propose strategic initiatives tailored to empower PAGs, thereby addressing the identified gaps and enhancing health equity in the region.

## 2 Methods

### 2.1 Participant selection

Participants (patient advocates) were selected based on their involvement and expertise in patient advocacy and cancer care within their respective countries (Table 1). The selection process aimed to ensure diverse representation from various regions and socioeconomic backgrounds within the MEA region.

**Table 1:** List of advocacy advisors who attended the meeting.

Sr. No.	Advisor name	Country	Title(s) & Organization(s)
1	Dr. Sawsan Al-Madhi*	UAE	Advocacy Advisor, CEO & Founder, AlignnEficient Health Consultancies
2	Ms. Lauren Pretorius	South Africa	Chief Executive Officer; Campaigning for Cancer
3	Dr. Emad Shash	Egypt	Head of Medical Oncology Department, Shefa Al Orman
4	Ms. Belma Kurdoglu	Türkiye	Chairman, Cancer Survivors Association
5	Dr. Ahmad Rabea	Egypt	Vice Head of the Department, Baheya Cancer Center
6	Dr. Atlal Abu Sanad	Saudi Arabia	Founder & Administrator, Najia community for survivors and patients with breast cancer
7	Mr. Hani Nassar	Lebanon	Barbara Nassar
8	Ms. Hamida Kettab	Algeria	El Amel Association
9	Ms. Benda Kithaka	Kenya	Executive Director and Founder; Kilele Health Association
10	Dr. Khaled Abdel Aziz	Egypt	Mersal Cancer Center

* Moderator for the meeting.

### 2.2 Meeting structure and agenda

The Health Equity Steering Committee for the MEA PAG convened a virtual meeting on July 26, 2023. The meeting agenda focused on discussing health equity from the perspective of PAGs at the MEA level. Discussions covered topics such as access to healthcare systems, the roles and challenges of patient organizations, and brainstorming on unmet needs and opportunities. The session ended with closing remarks and conclusions.

### 2.3 Data recording and analysis

The meeting was recorded to accurately capture the discussions. This recording was used internally for taking notes and analysis. The discussions were analyzed using the recorded content and notes. Recommendations were developed from the themes and consensus identified during the discussions. This included actionable steps to address the challenges and opportunities identified for improving health equity in the MEA region.

## 3 Results

The meeting was structured in three sections. First, it examined access to health-care systems in MEA, focusing on how easily patients can reach medical services, obtain appropriate medications, including innovative therapies, and receive holistic support at diagnosis, such as follow-up and counseling. Second, it reviewed the current practices and challenges of patient organizations, covering the services they provide, their disease-awareness efforts, and the need for capacity building. Lastly, a brainstorming session identified unmet needs and practical opportunities to improve health equity and patient-centered cancer care across the region.

### 3.1 Discussion 1: access to health care systems in MEA

A detailed discussion with the advocacy advisors revealed that access to medical facilities varies depending on geographical location and socioeconomic factors.

#### 3.1.1 Are patients able to access medical facilities regardless of where they live. E.g., remote or in a city?

##### 3.1.1.1 Geographical disparities in access to medical facilities

Patients in remote or rural areas often face challenges in accessing specialized treatment centers, including oncology centers for cancer care and may have limited access to comprehensive cancer management facilities, including chemotherapy, radiotherapy, and specialized surgeries. In Egypt, geographical access to medical facilities is challenging due to the country's large geographical area. Patients, particularly those from remote areas, face difficulties in accessing specialized cancer treatment centers (13). A similar situation was observed in south Africa (14). Türkiye also exhibits rural–urban disparities, with long waits and limited specialized services in rural public hospitals, similar to Egypt and South Africa. Distinctly, its dual public–private system creates additional divergence: privately insured patients can access a broader range of treatments more rapidly, whereas those relying on public insurance may have restricted access to newer, non-reimbursed products.

#### 3.1.2 Are there any financial barriers/issues that patients face when they want to obtain their medications?

##### 3.1.2.1 Financial constraints that contribute to disparities in access

Patients often struggle with transportation costs to access medical facilities in urban centers, particularly in remote areas. The absence of explicit discussion on travel insurance for medical costs in Egypt implies that it may not be a prevalent or widely available option for patients seeking healthcare services. Additionally, out-of-pocket expenses for treatments, medications, and supportive care services can pose significant financial burdens, particularly for patients from low socioeconomic backgrounds. In Lebanon, economic instability and currency devaluation have exacerbated access issues. Patients face challenges accessing essential medications, including cancer treatments, due to supply chain disruptions and financial constraints. Furthermore, insurance coverage plays a crucial role in determining access to medical facilities, particularly in Lebanon. Patients with private insurance coverage may have greater access to specialized treatments and facilities compared to those reliant on public healthcare systems. However, even with insurance, coverage limitations and high co-payments for certain medications and treatments can hinder access for patients. This disparity is particularly evident in Türkiye, where insurance status directly affects the accessibility and affordability of cancer care. Those with private insurance have access to all available treatment options. In contrast, patients with public insurance can access all standard treatments, but they cannot obtain products that are not yet on the market or reimbursed (15).

##### 3.1.2.2 Recommendations on how access to these facilities could be improved

Several potential solutions to improve access to medical facilities across all the countries were discussed

###### 3.1.2.2.1 Telemedicine and technology

Utilizing telemedicine and digital healthcare platforms can help bridge the gap in access to specialized care for patients in remote areas. Teleconsultations, remote monitoring, and teletherapy services can enhance access to medical expertise and support. Virtual online groups and communities designed to support and empower survivors and patients can help overcome cultural, geographical, and personal barriers. These platforms facilitate connections, sharing, and the exchange of experiences. However, supervision by healthcare providers is crucial to ensure the accuracy and quality of information exchanged. Dr. Abu Sanad highlights the successful establishment and maintenance of a secure and trusted online space for women with breast cancer, which has provided positive outcomes for over eight years (16). Remote symptom monitoring in palliative cancer care is gaining momentum in low-resource settings; for example, the PROSE intervention in sub-Saharan Africa enables side-effect reporting, clinical documentation, and the capture of patients' perspectives through a mobile phone application, thereby improving timely clinical decision-making and patient engagement (17).

###### 3.1.2.2.2 Infrastructure development

Investing in healthcare infrastructure and expanding the reach of medical facilities to underserved areas can improve access. Implementing regional healthcare initiatives to decentralize specialized services. Establishing mobile healthcare units to provide essential services, including comprehensive cancer screenings and treatment, in rural and remote regions. Evidence from Africa suggests that decentralization of cancer services through an integrated regional cancer centers not only increases access to care but also results in reductions in morbidity and mortality (18, 19).

###### 3.1.2.2.3 Financial support programs

Implementing financial support programs, such as subsidies for transportation costs and medication expenses, can alleviate the financial burden on patients and improve access to care. Implementing innovative financing mechanisms, such as patient assistance programs and drug subsidy schemes.

###### 3.1.2.2.4 Policy reforms

Advocating policy reforms to enhance insurance coverage, reduce out-of-pocket expenses, and ensure equitable distribution of healthcare resources can address systemic barriers to access.

#### 3.1.3 When it comes to holistic support around diagnosis (E.g., follow up with patients, patient counselling), is it provided by the health care system in your country? What additional provision could be added?

Several country-specific discussions highlight a range of challenges and strategies in providing holistic support for cancer patients, including addressing financial barriers, leveraging technology, advocating for policy changes, and fostering community collaboration (Table 2).

**Table 2:** Country-wise best practices in patient support programs.

Country	Best practices
**Egypt**	**Geographical access:** Mersal Cancer Center, focuses on providing complimentary drugs and establishing connections with doctors in various areas to ensure patients receive necessary care without traveling long distances. **Telemedicine:** Mersal Cancer Center, utilizes a business website system for telecommunication and patient support. **Government initiatives:** Efforts are made to integrate universal health insurance, starting with areas such as Luxor, to improve access to medical facilities.
**South Africa**	**Transportation support:** Efforts are made to provide transportation for patients living far from treatment facilities, especially in rural areas. **Community support:** Patients receive support from patient groups and civil society organizations, including accommodation and legal assistance. **Policy advocacy:** Lobbying efforts aim to include private healthcare in co-payment programs and challenge decisions that restrict access to treatment.
**Kenya**	**Stigma and knowledge barriers:** Efforts are made to address stigma and improve knowledge dissemination about cancer and available support programs. **Technology use:** patient organizations use technology to record and share knowledge, ensuring institutionalization of information and advocacy efforts. **Regional collaboration:** Knowledge and best practices are shared across Africa to support advocacy and improve patient outcomes.
**Türkiye**	**Advocacy efforts:** Patient organizations work together to inform the government about patient needs and to advocate for better coverage of treatments, especially targeted therapies, by demonstrating their long-term benefits. **Holistic care:** Discussions revolve around the need for a multidisciplinary approach to cancer care and the importance of including various services such as palliative care and mental health (psychological) support in insurance coverage.
**Lebanon**	**Government support:** Efforts are made to improve drug distribution systems and ensure uninterrupted access to medications, although financial constraints remain a significant barrier.

### 3.2 Discussion 2: current practices of patient organizations and challenges

#### 3.2.1 What do the current health services/support that patient organizations provide for the patients?

In South Africa, Ms. Pretorius's organization initiated a platform for patient advocates to share educational materials and knowledge across the region. This initiative aims to ensure that relevant learning materials are available to new advocates, fostering better-informed patient advocacy. They emphasize the importance of creating locally relevant educational materials, especially regarding non-communicable diseases, to improve health literacy and address specific country contexts.

In Algeria, as the chair of a national committee on cancer prevention, Ms. Kettab's organization provides various essential services including mental and physical health support, legal rights awareness, and extensive awareness campaigns. They started the first solidarity program for companionship and mental/physical health in Algeria. Ms. Kettab's organization educates patients about their rights under the Algerian constitution, empowering them to advocate for themselves and access the care they need. They also engage with religious leaders to address cultural sensitivities surrounding cancer, such as reluctance to undergo certain treatments or examinations. Furthermore, to combat stigma and raise awareness about cancer, Ms. Kettab's organization conducts extensive awareness campaigns which include initiatives such as the “Hope Convoy,” which travels to various cities and remote areas to educate communities about cancer prevention, early detection, and available resources.

In Kenya, Ms. Kithaka's organization focuses on empowering cancer patients and survivors with knowledge and skills to navigate their cancer journey effectively. They provide education and training programs to help patients understand their diagnosis, treatment options, and rights within the healthcare system. Ms. Kithaka's organization conducts extensive awareness campaigns to educate the public about the signs and symptoms of cancer, promote healthy behaviors, and encourage regular screenings. By raising awareness at the grassroots level, they empower communities to take proactive steps towards cancer prevention and early intervention. Ms. Kithaka advocates for investments in organizational development and capacity-building initiatives by providing training opportunities for cancer advocates, healthcare professionals, and community leaders, equipping them with the skills and resources needed to support cancer patients. By building the capacity of local organizations, they strengthen the overall cancer care ecosystem and improve outcomes for patients.

Mr. Nassar's organization in Lebanon offers guidance, support, and resources to cancer patients and their families. They provide practical assistance such as wigs for women experiencing hair loss due to treatment and organize awareness events. Moreover, they advocate for patients' rights, including access to treatments, and work with hospitals across Lebanon to ensure comprehensive support for cancer patients.

In Egypt, Dr. Shash's organization focuses on correcting misconceptions about cancer through accessible educational materials. They employ graphic content and Arabic language materials, validated by medical professionals, to ensure effective communication. Furthermore, they offer comprehensive patient education programs covering various aspects of cancer care, including communication skills, nutrition counseling, and psychological support.

Patient organizations in Türkiye, including the Cancer Survivors Association, regularly conduct training sessions and public awareness campaigns. Dr. Kurdoglu notes that they collaborate with the Medical Oncology Society and use social media, television, and celebrity endorsements to engage a broader audience. These educational efforts aim to enhance awareness and understanding of cancer issues. Additionally, their awareness campaigns not only raise disease awareness but also facilitate patient screening, enabling early detection through these initiatives.

*Gaps to be addressed:*

• While patient organizations gather data, there's a gap in transforming this data into actionable knowledge.
• Many patient organizations lack the necessary resources and expertise to build capacity effectively.
• Collaboration among patient organizations, healthcare professionals, government agencies, and private sector entities is often limited.
• Patient organizations face challenges in accessing resources such as funding, infrastructure, and expertise.

#### 3.2.2 How do patient organizations update their information regarding a specific type of tumor? Are there specific resources? How is disease awareness in the area disseminated to the public?

Patient organizations across the participating countries employed a multifaceted approach, including knowledge sharing, community engagement, advocacy, collaboration, and leveraging technology, to update information, raise awareness, and support individuals affected by cancer (Table 3).

**Table 3:** Patient organizations strategies to update information on types of tumors and disseminate awareness to the public.

Country	Strategies
**South Africa**	**Patient advocates incubator:** Initiatives such as the Patient Advocates Incubator serve as a centralized platform for sharing educational materials among patient advocates. **Localized educational materials:** Efforts are made to create educational resources in multiple languages and adapt them to the local context, addressing linguistic and cultural diversity.
**Algeria**	**Solidarity programs and awareness campaigns**: Implementation of solidarity programs to address the holistic needs of cancer patients, including mental health support, rehabilitation, and legal rights awareness. “**Hope convoy”**: Awareness campaigns such as the “Hope Convoy” includes doctors who are specialists in cancer and religious leaders visiting various cities to raise awareness about cancer, particularly targeting underserved communities.
**Lebanon**	**Patient support and advocacy:** Organizations such as Barbara Nassar's provide essential guidance and support services to cancer patients and their families, including access to accessories such as wigs, advocacy for treatment access, and awareness initiatives. Efforts to advocate for access to specific drugs such as Olaparib for ovarian cancer patients, highlighting the importance of ensuring that patients receive appropriate and timely treatment options. **Cancer Awareness Villages and Educational Events:** Events such as the cancer awareness village and educational meetings engage multiple stakeholders, including university hospitals, universities, and NGOs, to raise awareness and provide information to the public.
**Egypt**	**Patient education and misinformation correction**: Initiatives focus on correcting misconceptions and misinformation about cancer through educational programs, utilizing graphics and Arabic-language materials to improve accessibility and understanding. **Professional-led social media groups**: Professional-led social media groups provide a platform for patients to access accurate information, share experiences, and receive support from trained moderators and peers.
**Türkiye**	**Use of social media and celebrity endorsements:** Social media platforms, along with celebrity endorsements and TV programs, are utilized to disseminate information and raise awareness about cancer, reaching a broader audience.

**Table 4:** Summary of unmet needs and opportunities.

Discussion point	Key insights
Involvement in decision making	Patient organizations like those in Algeria, Lebanon, and Türkiye are deeply involved in decision-making that influences cancer care, advocating for improved services and policy changes. These efforts include everything from mental health support to the establishment of new cancer centers and public awareness campaigns.
Planned cancer care projects	Organizations are planning diverse projects focusing on education, awareness, and palliative care. These include training, media campaigns, and direct patient support initiatives, as seen in Türkiye and Egypt, aiming to enhance patient knowledge and treatment access.
Barriers in achieving equitable healthcare	The most significant barriers identified include inequitable access to innovative treatments and financial constraints. Experts like Dr. Abdel Aziz and Dr. Kurdoglu emphasize the need for better collaboration across sectors and countries to improve drug access and integrate patients in care planning, stressing on financial models and policy advocacy.

#### 3.2.3 Do you believe that the current resources are reaching the right patient in the right format? (Wi-Fi connections, understanding levels, language barriers)

While there are ongoing efforts to provide resources and support to cancer patients, the discussion among the panelist suggests that there are still gaps and challenges in ensuring that these resources reach the right patients in the right format.

• Traditional methods of information dissemination, such as brochures, may not be as effective as they once were, indicating a need for alternative approaches to spread information.
• Not all patients have equal access to essential resources such as diagnostics and oncology services, innovative medicines/reimbursement, transportation and lodging, reliable internet/phone connectivity, and culturally/linguistically appropriate information and counseling
• While social media platforms have potential for offering support and disseminating information, there are challenges of monitoring and verifying information on these platforms indicate that further efforts may be necessary to guarantee the accuracy and reliability of online resources.

Addressing these challenges may require innovative approaches, increased patient involvement, and continued investment in capacity building and information dissemination efforts.

#### 3.2.4 Is your patient organization engaged with a third party to enhance your capabilities? Is this area something that you're interested in, and what kind of capabilities you might need?

Patient organizations across these participating countries demonstrated a commitment to collaborate with third parties to enhance their capabilities in supporting cancer patients. They identify areas such as capacity building, financial support, access to expertise, and resource allocation as essential for strengthening their initiatives and achieving their goals.

### 3.3 Discussion 3: brainstorming session—identifying unmet needs and opportunities

#### 3.3.1 Are patient organizations involved in decision making that would change the current practice in the area?

In Algeria, Ms. Kettab chairs a national committee on cancer prevention, representing 18 cancer societies. Their efforts focus on complementary cancer care services, including mental health support, rehabilitation, and legal rights awareness for cancer patients. They collaborate with policymakers, healthcare professionals, and other stakeholders to advocate for changes that improve cancer care delivery and patient outcomes. Similarly, in Lebanon, Mr. Nassar's organization collaborates with hospitals, universities, and NGOs to provide comprehensive support to cancer patients and advocate for policy changes. Recently, they received approval to build the first adult cancer center in Lebanon, emphasizing essential services such as free clinics, palliative care, and awareness programs. In Türkiye, by performing awareness campaigns and conducting special projects for unmet needs of cancer patients, we become voice of cancer patients and increase the awareness of unmet needs of cancer patients in Türkiye.

#### 3.3.2 Do you have planned cancer care projects in the next one or two years? Could you describe it further?

In Türkiye, patient organizations conduct regular training meetings, educational programs, and awareness campaigns in collaboration with associations, universities, and healthcare professionals. They utilize social media and traditional media channels to disseminate information and engage patients. Beside those, one of the main issues that all NGOs working with cancer patients is focusing on palliative care in Türkiye. In Egypt, efforts are focused on correcting misinformation and providing comprehensive educational programs for cancer patients and caregivers. These projects include patient education initiatives, capacity building workshops, and advocacy campaigns aimed at improving access to treatment and support services.

#### 3.3.3 Based on the discussion today, what do you believe is the biggest barrier in achieving equitable health care provision and how can we work on that together?

The biggest barrier identified in achieving equitable healthcare provision is the lack of equitable access to innovative drugs, as highlighted by Dr. Abdel Aziz. To address this barrier, collaboration among patient organizations and other NGOs is essential. Dr. Abu Sanad emphasizes the importance of expanding compassionate release programs and engaging patients in treatment planning and survivorship support. Rehabilitation programs are also identified as weak across the Middle East, hindering the reintegration of cancer survivors into society. Dr. Kurdoglu mentioned that the biggest barrier in achieving equitable health care provision is accessing new generation products which are not reimbursed in Türkiye. Financial constraints pose significant challenges, requiring innovative financial models and collaboration with governmental and non-governmental entities. Capacity building, data sharing, and knowledge exchange are crucial for overcoming barriers and achieving equitable cancer care provision. Collaboration among patient organizations, ministries/payers, hospitals and centers of excellence, industry, academia, and community/faith groups, across organizations and countries can help leverage resources and advocate policy changes to improve financial sustainability and access to cancer care services.

## 4 Discussion

This steering committee meeting consensus was an endeavor, aiming to explore the complex issues surrounding health equity and patient advocacy in cancer care within the MEA region. By convening PAG experts across various countries, healthcare settings, and perspectives across the MEA, the meeting facilitated a comprehensive examination of patient-centric care fostering a broad and inclusive discussion.

### 4.1 Key findings from discussion sessions

Three main themes emerged from the discussions:

1. **Collaborations**: The participants highlighted the necessity for extensive collaborations between patient organizations, ministries/payers, hospitals/centers of excellence, academia, industry, media, and community/faith groups. These partnerships are crucial for enhancing knowledge sharing, gaining access to the latest treatment advancements, and combating societal stigma associated with diseases through effective community engagement.
2. **Data and scalability**: There was a consensus on the significant gap in data handling by patient organizations, particularly in collecting and utilizing real-world data to gauge and enhance the effectiveness of access programs. This underscores a critical need for these organizations to strengthen their data analytics capabilities.
3. **Sustainability and prevention**: Sustainability emerged as a critical yet often neglected area. Discussions emphasized that preventive measures are not only more cost-effective but also sustainable long-term strategies. The potential of artificial intelligence (AI) to revolutionize health equity was also a topic of interest, suggesting an avenue for future technological integration.

The most significant barrier to achieving equitable healthcare identified in the discussions is the restricted access to innovative and effective drug treatments. The panel discussions revealed that access to medical facilities in the MEA region varies significantly based on geographical location and socioeconomic status, echoing global findings on health disparities. Geographical challenges, particularly in rural areas, limit access to specialized cancer care, including treatments like chemotherapy and radiotherapy, a scenario that aligns with studies conducted in similar settings globally (5, 20, 21). Financial barriers further exacerbate these disparities, with patients facing high out-of-pocket expenses and inconsistent insurance coverage, which is a recurring theme in health equity research (22). Enhancing infrastructure, adopting telemedicine, and reforming policies to increase insurance coverage could mitigate these disparities (23). In the MEA region, collaboration across different organizations and countries is vital to leverage resources effectively and advocate for policy changes that will ensure financial sustainability and broader access to cancer care.

Furthermore, the committee discussions highlighted a significant gap in the handling and utilization of real-world data by patient organizations. This observation underscores the need for better data analytics capabilities such as shared minimum data set, simple dashboards and routine data-quality checks to enhance the effectiveness of access programs, which aligns with recent findings on the importance of data in health equity (24). Building robust data analytics frameworks within patient organizations could enable more precise targeting of health interventions and resource allocation (25). Short, skills-based training in data analysis could be a practical step forward.

Lastly, the discussions around sustainability and the emphasis on prevention reflect a strategic shift towards long-term health planning, which is crucial in cancer management. This aligns with global trends where preventative health measures are increasingly recognized for their cost-effectiveness and broader health benefits (26–29). There is a need for increased investment in preventative health programs and the integration of AI technologies to predict health trends and personalize patient care (30, 31). Collaborative efforts should focus on developing sustainable health programs that include community-based preventive measures.

In May 2024, the World Health Assembly adopted a resolution reinforcing the importance of social participation for universal health coverage (UHC) and well-being. The resolution emphasizes empowering individuals, communities, and civil society to participate inclusively in decision-making processes across all levels of the health system. This approach ensures that policies and programs are more responsive to the needs of vulnerable and marginalized groups, fostering greater equity, transparency, and trust in healthcare systems. The resolution also highlights the critical need for regular and sustained social participation, backed by adequate resources, to advance health equity and promote resilience in the face of health emergencies. This aligns with global efforts to address social determinants of health and tackle widening inequities exacerbated by the COVID-19 pandemic, climate change, and other global crises (32, 33).

Overall, to improve health equity in the oncology within the MEA region, the particpants of the steering committee suggested a multi-faceted approach that addresses the challenges faced by the MEA region. First, it's crucial to empower patient organizations to participate more actively in decision-making and policy development, particularly in designing patient support programs and developing screening initiatives. Collaborative projects should be expanded to foster data sharing and joint efforts across borders, particularly for accessing innovative but non-reimbursed drugs. Innovative financial models need to be developed to reduce the economic burden on patients, while educational programs should be amplified to empower patients throughout their treatment journey. There is also a need for better rehabilitation programs and legal support for patients to ensure comprehensive care and effective reintegration into society post-treatment. Leveraging technology, such as telemedicine, can extend healthcare reach, especially in underserved areas. Enhanced data collection and utilization are critical for effective resource allocation and advocacy for policy reform. Community involvement must be intensified through awareness campaigns and promoting early detection and healthy lifestyles. Finally, ensuring financial sustainability through expanded insurance coverage and innovative funding mechanisms will be key to maintaining and advancing cancer care provisions in the region.

## 5 Conclusion

The discussion from the PAGs from the MEA region highlights the existing challenges and disparities in cancer care accessibility, influenced by geographic and socioeconomic factors. Patient organizations are actively involved in decision-making, enhancing policy and treatment access, yet challenges persist, notably in equitable access to innovative treatments and financial constraints impacting patient care. The call to action emphasizes the need for stronger collaborations across patient organizations, government bodies or policy makers, and the pharmaceutical industry to address these disparities. Initiatives should focus on integrating patient perspectives into treatment planning and expanding innovative drug access through consistent regional strategies. Future directions must focus on enhancing data collection mechanisms to inform policy and practice, enhancing patient education, and leveraging technology to streamline cancer care. Developing comprehensive cancer registries and improving financial support mechanisms will be pivotal. Additionally, capacity building within patient organizations and continuous policy advocacy will also be essential to foster a more equitable healthcare landscape across the MEA region.

## Data Availability

The raw data supporting the conclusions of this article will be made available by the authors, without undue reservation.

## References

[1] World Health Organization Health equity. Geneva, Switzerland (2024). Available online at: https://www.who.int/health-topics/health-equity#tab=tab_1 (Accessed November 2024).

[2] Braveman P Gruskin S. Defining equity in health. J Epidemiol Community Health. (2003) 57(4):254–8. 10.1136/jech.57.4.254 12646539 PMC1732430

[3] Hofmarcher T Manzano Garcia A Wilking N Lindgren P. The disease burden and economic burden of cancer in 9 countries in the Middle East and Africa. Value Health Reg Issues. (2023) 37:81–7. 10.1016/j.vhri.2023.05.005 37364406

[4] Tuck CZ Cooper R Aryeetey R Gray LA Akparibo R. A critical review and analysis of the context, current burden, and application of policy to improve cancer equity in Ghana. Int J Equity Health. (2023) 22(1):254. 10.1186/s12939-023-02067-2 38066530 PMC10709985

[5] Mansour R Abdel-Razeq H Al-Hussaini M Shamieh O Al-Ibraheem A Al-Omari A Systemic barriers to optimal cancer care in resource-limited countries: Jordanian healthcare as an example. Cancers (Basel). (2024) 16(6):1117. 10.3390/cancers16061117 38539452 PMC10968872

[6] Brand NR Qu LG Chao A Ilbawi AM. Delays and barriers to cancer care in low- and middle-income countries: a systematic review. Oncologist. (2019) 24(12):e1371–80. 10.1634/theoncologist.2019-0057 31387949 PMC6975966

[7] Hoos A Anderson J Boutin M Dewulf L Geissler J Johnston G Partnering with patients in the development and lifecycle of medicines: a call for action. Ther Innov Regul Sci. (2015) 49(6):929–39. 10.1177/2168479015580384 26539338 PMC4616907

[8] Robbins DA Curro FA Fox CH. Defining patient-centricity: opportunities, challenges, and implications for clinical care and research. Ther Innov Regul Sci. (2013) 47(3):349–55. 10.1177/2168479013484159 30231434

[9] Tolotti A Barello S Vignaduzzo C Liptrott SJ Valcarenghi D Nania T Patient engagement in oncology practice: a qualitative study on patients’ and nurses’ perspectives. Int J Environ Res Public Health. (2022) 19(18):11644. 10.3390/ijerph191811644 36141919 PMC9517681

[10] Bellomo C. Patient advocacy/patient empowerment. J Oncol Navig Surviv. (2016) 7(8).28845364

[11] Alkhaibari RA Smith-Merry J Forsyth R Raymundo GM. Patient-centered care in the Middle East and North African region: a systematic literature review. BMC Health Serv Res. (2023) 23(1):135. 10.1186/s12913-023-09132-0 36759898 PMC9909864

[12] The Academy of Medical Sciences. Advancing universal health coverage in the Middle East and North Africa: The role of research (2020). Available online at: https://acmedsci.ac.uk/file-download/19558481 (Accessed November 2024).

[13] Radwan G Adawy A. The Egyptian health map: a guide for evidence-based decisionmaking. East Mediterr Health J. (2019) 25(5):350–61. 10.26719/emhj.18.048 31364760

[14] Willie MM. Analysis of cancer care services: a case study approach in the Eastern Cape Province, South Africa. Adv Healthcare Res. (2025) 3(1):60–8. 10.60079/ahr.v3i1.461

[15] Kesici Z Yilmaz V. Insurance-based disparities in breast cancer treatment pathways in a universal healthcare system: a qualitative study. BMC Health Serv Res. (2023) 23(1):112. 10.1186/s12913-023-09108-0 36732811 PMC9894738

[16] Abusanad A. “Najia” story: a WhatsApp support group for patients with breast cancer. Innov Digit Health Diagn Biomark. (2020) 1(1):16–8. 10.36401/iddb-20-01

[17] Salako O Enyi A Miesfeldt S Kabukye JK Ngoma M Namisango E Remote symptom monitoring to enhance the delivery of palliative cancer care in low-resource settings: emerging approaches from Africa. Int J Environ Res Public Health. (2023) 20(24):7190. 10.3390/ijerph20247190 38131741 PMC10743024

[18] Nyangasi MF McLigeyo AA Kariuki D Mithe S Orwa A Mwenda V. Decentralizing cancer care in Sub-Saharan Africa through an integrated regional cancer centre model: the case of Kenya. PLOS Glob Public Health. (2023) 3(9):e0002402. 10.1371/journal.pgph.0002402 37738236 PMC10516416

[19] Das M. Decentralisation to improve cancer services in DR Congo. Lancet Oncol. (2022) 23(4):457. 10.1016/S1470-2045(22)00129-2 35248188

[20] George M Smith A Ranmuthugula G Sabesan S. Barriers to accessing, commencing and completing cancer treatment among geriatric patients in rural Australia: a qualitative perspective. Int J Gen Med. (2022) 15:1583–94. 10.2147/IJGM.S338128 35210830 PMC8859537

[21] Joensen BM Nielsen S Roin A. Barriers to quality of care for cancer patients in rural areas: a study from the Faroe Islands. J Multidiscip Healthc. (2020) 13:63–70. 10.2147/JMDH.S233313 32021235 PMC6974407

[22] Riley WJ. Health disparities: gaps in access, quality and affordability of medical care. Trans Am Clin Climatol Assoc. (2012) 123:167–72; discussion 172–4. 23303983 PMC3540621

[23] Khoong EC. Policy considerations to ensure telemedicine equity. Health Aff (Millwood). (2022) 41(5):643–6. 10.1377/hlthaff.2022.00300 35500190

[24] Chisolm DJ Dugan JA Figueroa JF Lane-Fall MB Roby DH Rodriguez HP Improving health equity through health care systems research. Health Serv Res. (2023) 58(Suppl 3):289–99. 10.1111/1475-6773.14192 38015859 PMC10684038

[25] Dash S Shakyawar SK Sharma M Kaushik S. Big data in healthcare: management, analysis and future prospects. J Big Data. (2019) 6(1):54. 10.1186/s40537-019-0217-0

[26] Thun MJ DeLancey JO Center MM Jemal A Ward EM. The global burden of cancer: priorities for prevention. Carcinogenesis. (2010) 31(1):100–10. 10.1093/carcin/bgp263 19934210 PMC2802672

[27] Cheatley J Aldea A Lerouge A Devaux M Vuik S Cecchini M. Tackling the cancer burden: the economic impact of primary prevention policies. Mol Oncol. (2021) 15(3):779–89. 10.1002/1878-0261.12812 33021030 PMC7931126

[28] Mansour R Al-Ani A Al-Hussaini M Abdel-Razeq H Al-Ibraheem A Mansour AH. Modifiable risk factors for cancer in the Middle East and North Africa: a scoping review. BMC Public Health. (2024) 24(1):223. 10.1186/s12889-024-17787-5 38238708 PMC10797965

[29] Naja F Nasreddine L Awada S El Sayed Ahmad R Hwalla N. Nutrition in the prevention of breast cancer: a Middle Eastern perspective. Front Public Health. (2019) 7:316. 10.3389/fpubh.2019.00316 31788465 PMC6856137

[30] Davenport T Kalakota R. The potential for artificial intelligence in healthcare. Future Healthc J. (2019) 6(2):94–8. 10.7861/futurehosp.6-2-94 31363513 PMC6616181

[31] Johnson KB Wei WQ Weeraratne D Frisse ME Misulis K Rhee K Precision medicine, AI, and the future of personalized health care. Clin Transl Sci. (2021) 14(1):86–93. 10.1111/cts.12884 32961010 PMC7877825

[32] Boivin A Mothci D Dumez V Shore F Bok A. World leaders unite to embed social participation in health systems. Br Med J. (2024) 386:q1460. 10.1136/bmj.q1460 38986548

[33] World Health Organization. Social participation for universal health coverage, health and well-being (2024). Available online at: https://apps.who.int/gb/ebwha/pdf_files/EB154/B154_CONF10-en.pdf (Accessed November 2024).

